# The origin of efficient triplet state population in sulfur-substituted nucleobases

**DOI:** 10.1038/ncomms13077

**Published:** 2016-10-05

**Authors:** Sebastian Mai, Marvin Pollum, Lara Martínez-Fernández, Nicholas Dunn, Philipp Marquetand, Inés Corral, Carlos E. Crespo-Hernández, Leticia González

**Affiliations:** 1 Institute of Theoretical Chemistry, Faculty of Chemistry, University of Vienna, Währinger Straße 17, Vienna 1090, Austria; 2 Center for Chemical Dynamics and Department of Chemistry, Case Western Reserve University, 10900 Euclid Avenue, Cleveland, Ohio 44106, USA; 3 Universidad Autónoma de Madrid, Departamento de Química, Cantoblanco, Madrid 28049, Spain

## Abstract

Elucidating the photophysical mechanisms in sulfur-substituted nucleobases (thiobases) is essential for designing prospective drugs for photo- and chemotherapeutic applications. Although it has long been established that the phototherapeutic activity of thiobases is intimately linked to efficient intersystem crossing into reactive triplet states, the molecular factors underlying this efficiency are poorly understood. Herein we combine femtosecond transient absorption experiments with quantum chemistry and nonadiabatic dynamics simulations to investigate 2-thiocytosine as a necessary step to unravel the electronic and structural elements that lead to ultrafast and near-unity triplet-state population in thiobases in general. We show that different parts of the potential energy surfaces are stabilized to different extents via thionation, quenching the intrinsic photostability of canonical DNA and RNA nucleobases. These findings satisfactorily explain why thiobases exhibit the fastest intersystem crossing lifetimes measured to date among bio-organic molecules and have near-unity triplet yields, whereas the triplet yields of canonical nucleobases are nearly zero.

## Abstract (web summary)

Sulfur-substituted nucleobases are promising photo- and chemotherapeutic drugs. Here, the authors unravel the electronic and structural aspects that lead to the ultrafast population of triplet states in these molecules, providing an explanation for their efficiency as photosensitizers.

## Graphical abstract

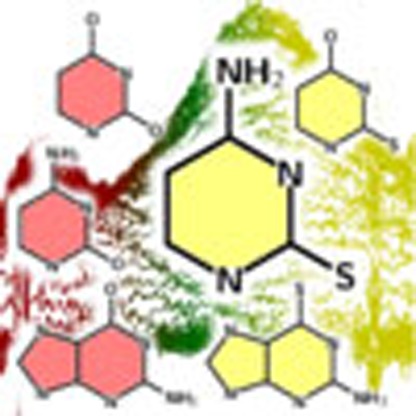

Chemically modified nucleobases provide DNA and, in particular, RNA with additional functions that are only beginning to be discovered^1^. Thiobases, where the oxygen atom of a carbonyl group is substituted by a sulfur atom, have been detected in transfer-RNA^2^,^3^, and the lack of some of these substituted nucleobases can lead to metabolic diseases in the mitochondria^4^. Thiobases are also employed in DNA-based nanotechnology^5^, in photocrosslinking studies^6^,^7^, in pharmacology^8^,^9^, and—due to their interesting photophysical properties—in photo-chemotherapy^10^,^11^,^12^. In fact, thiobases have recently been demonstrated as effective photo-chemotherapeutic agents for the treatment of skin cancer in tissue biopsies^13^ as well as of bladder cancer in animal models^11^, and are being developed for clinical applications^14^.

The canonical nucleobases absorb primarily in the ultraviolet-C range (100–280 nm). In contrast, substitution of the oxygen atom by sulfur induces a significant red-shift in the absorption spectrum, making thiobases effective absorbers of ultraviolet-B (280–315 nm) and ultraviolet-A (315–400 nm) radiation^15^. An intriguing observation is that, while canonical nucleobases efficiently return to the electronic ground state following electronic excitation^16^,^17^, thiobases populate long-lived, reactive triplet states in near-unity yields^15^—a property that is exploited in their photonic applications. The photostability of canonical nucleobases is ascribed to ultrafast relaxation mechanisms that convert the photon energy into vibrational motion^17^. Although intense research activity has been aimed at determining time scales and relaxation pathways in thiobases by using time-resolved spectroscopic techniques^12^,^18^,^19^,^20^,^21^,^22^,^23^,^24^ and theoretical methods^25^,^26^,^27^,^28^,^29^,^30^,^31^,^32^, there exists no physical explanation of the electronic and structural factors that favour the formation of reactive triplet states instead of ultrafast relaxation to the ground state.

In this work, we provide a rationale for the origin of efficient population of triplet states in thiobases versus the ultrafast recovery of the ground state in canonical nucleobases. We use 2-thiocytosine (2tC) as a testbed to understand how thionation leads to a strong stabilization of different parts of the excited-state potential energy surfaces (PESs), which favours intersystem crossing (ISC) to the triplet manifold over internal conversion to the electronic ground state. Not only is 2tC specifically relevant for many medicinal applications—due to its anti-cancer^33^, anti-viral^34^, anti-bacterial^35^, anti-microbial^36^ and cytotoxic activities^37^—but it is the only pyrimidine thiobase derivative that has not yet been investigated using time-resolved techniques^15^. By filling this gap we are able to deliver a comprehensive explanation of the remarkable photophysics of thiobases, providing design criteria for the synthesis of prospective drugs.

## Results

### Photochemical mechanism of 2-thiocytosine

**Figure 1:**
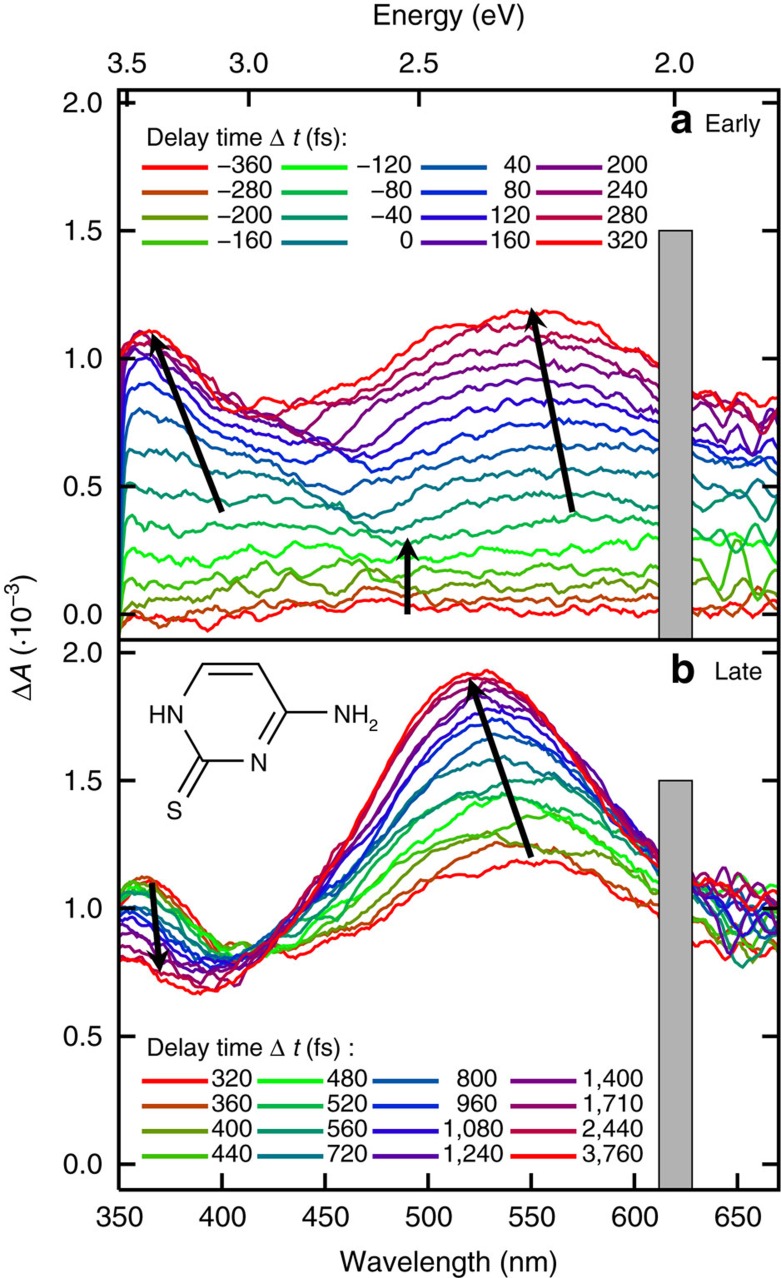
Transient absorption spectra. (**a**) Early time delays (−360 to 320 fs) and (**b**) later time delays (320 to 3,760 fs). The spectra were recorded in phosphate buffer solution (pH 7.4) using an excitation wavelength of 308 nm. Arrows indicate the movement of the absorption bands with time. The grey boxes block the overtone band of the excitation light. The inset shows the molecular structure of 2-thiocytosine.

A combination of steady-state and femtosecond transient absorption spectroscopy with ab initio computations and nonadiabatic dynamics is used to elucidate the excited-state dynamics of 2tC. Femtosecond transient absorption spectra (fs-TAS) were recorded exciting with UV-B (308 nm) and UV-A (321 nm) wavelengths, which correspond to the low-energy tail of the steady-state absorption spectrum (see Supplementary Fig. 1). According to calculations (see Supplementary Fig. 2, Supplementary Table 1 and Supplementary Note 1), the UV-A or UV-B wavelengths excite the lowest-energy ^1^*π*_*s*_π* state (the *S*_2_ at the equilibrium geometry) or the tail of the second ^1^*π*_*s*_π* state (*S*_4_), respectively. The TAS obtained after 308 nm excitation (Fig. 1, the TAS exciting at 321 nm is shown in Supplementary Fig. 3) shows an initial featureless rise in the absorbance covering the full spectral probe window from −360 fs until a time delay of −120 fs (that is, within the cross correlation of the pump and probe beams), when two distinct absorption maxima begin to emerge: one in the UV range (350–400 nm) and another in the visible range (around 580 nm). These absorption bands continue to grow until 320 fs, at which point the UV band reaches its maximum. As the UV band decays, the visible band continues to increase in amplitude and blueshifts to *λ*_max_=525 nm at 3.76 ps, with an apparent isosbestic point at 420 nm. After 3.76 ps, no significant changes in the absorption maxima are observed for a time delay of up to 20 ps (not shown), the maximum time delay investigated in this work.

**Figure 2:**
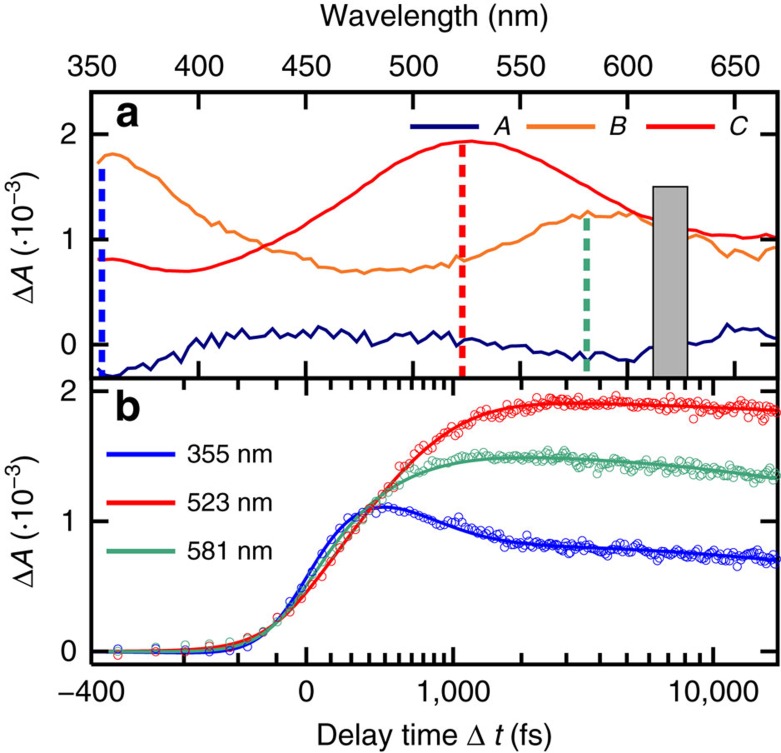
Target and global analysis of the TAS. (**a**) Decay associated spectra obtained by globally fitting the transient absorption data to a two-component sequential kinetic model (*A* and *B*) with a constant offset (*C*). (**b**) Kinetic traces and fitted functions from the global fit analysis, taken at representative probe wavelengths, as indicated with coloured dashed lines in (**a**).

The TAS data were subjected to a target and global fitting analysis. Two lifetimes (*τ*_1_=210±50 fs and *τ*_2_=480±60 fs) were needed to model the dynamics during the first 4 ps. Figure 2 shows the decay-associated spectra (DAS) and the fitted representative kinetic traces taken at the transient absorption maxima, that is, 355, 523 and 581 nm (see Supplementary Fig. 4 for the traces exciting at 321 nm).

**Figure 3:**
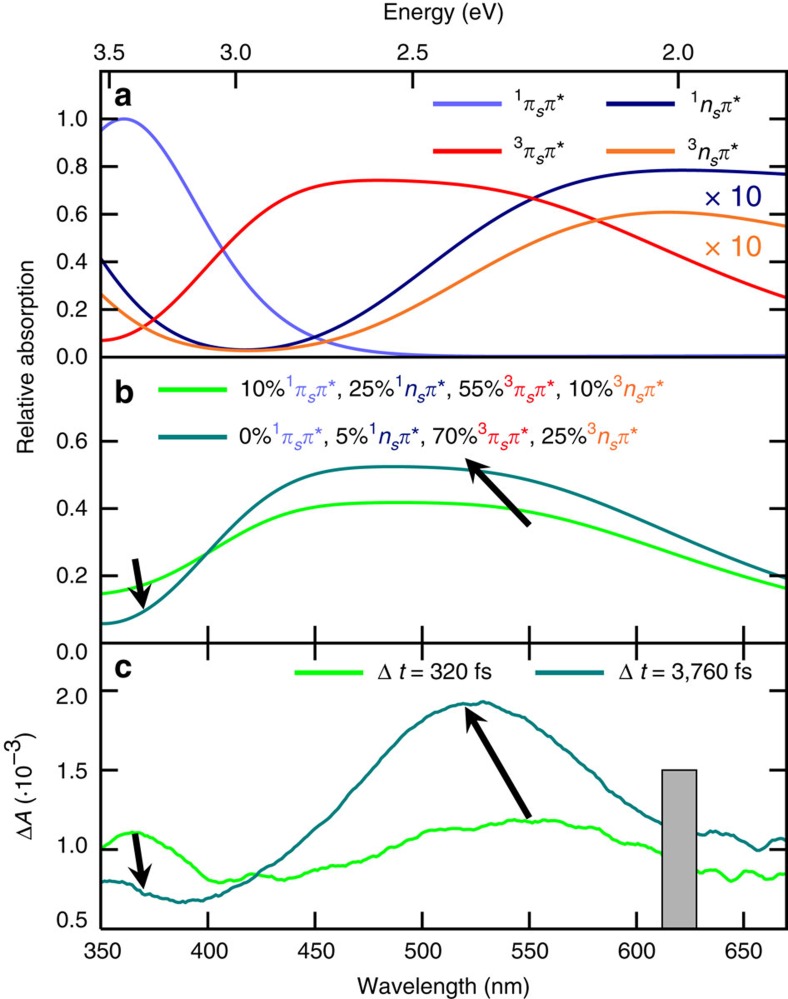
Simulated absorption spectra of the excited states. (**a**) Calculated absorption spectra from the given excited states at their respective minimum geometries (MS-CASPT2, convoluted using Gaussians with 0.7 eV full-width at half-maximum). (**b**) Linear combinations of the calculated spectra with the given proportions. (**c**) The experimental transient absorption spectra at the indicated time delays.

In order to interpret the TAS, and to deduce the relaxation pathways associated with the obtained lifetimes, the deactivation pathways out of the initially excited *S*_2_ (^1^*π*_*s*_π*) state were calculated using the MS-CASPT2 method (multi-state complete active space second-order perturbation theory). TAS were simulated (Fig. 3a, the corresponding data is given in Supplementary Table 2) from the excited-state minima (^1^*π*_*s*_π*, ^1^*n*_*s*_π*, ^3^*n*_*s*_π* and ^3^*π*_*s*_π*) using a sum of Gaussians centred at the calculated transition energies, with the amplitudes proportional to the oscillator strengths. The ^1^*π*_*s*_π* state absorbs strongly around 363 nm and the ^3^*π*_*s*_π* state dominates the visible part of the spectrum with two transitions around 441 and 546 nm, giving rise to a broad absorption band. In contrast, the two *n*_*s*_π* states show a 10-fold weaker absorbance compared with the *π*_*s*_π* states. The absorption spectra of the singlet and triplet *n*_*s*_π* states are very similar, with transitions at 333 and 576 nm versus 333 and 574 nm, respectively.

Figure 3b shows two linear combinations of the four calculated transient spectra harmonizing with the experimental transient spectra at selected time delays, that is, at 320 fs (when the UV band reaches its maximum intensity) and 3,760 fs (when the visible band reaches its maximum). The per cent contribution of each excited-state absorption can readily explain both the decay in the UV region (<400 nm) and the increase of the visible band around 525 nm. For comparison, the two corresponding experimental transient spectra are shown in Fig. 3c.

According to the calculations, the initial broad and featureless increase in absorption amplitude across the full spectral probe window until −120 fs (see also DAS *A* in Fig. 2a) can be assigned to a linear combination of the singlet states with *π*_*s*_π* and *n*_*s*_π* characters. This assignment is further supported by dynamics simulations, which predict that a ^1^*π*_*s*_π*/^1^*n*_*s*_π* conical intersection (CoIn) can be reached within 160 fs upon excitation, from which the molecule populates simultaneously two singlet minima with ^1^*n*_*s*_π* and ^1^*π*_*s*_π* characters.

At a time delay of about −80 fs, two distinct absorption bands begin to evolve, with maxima around 360 and 550 nm, respectively (Fig. 1 and DAS *B* in Fig. 2a). These absorption bands are assigned to a linear combination of the absorption from the ^1^*π*_*s*_π*, ^1^*n*_*s*_π*, ^3^*n*_*s*_π* and ^3^*π*_*s*_π* states (Fig. 3b). The UV absorption band matches most closely the spectrum computed for the ^1^*π*_*s*_π* state, thus the subsequent decay in this region is likely associated with a decay of ^1^*π*_*s*_π* population.

Finally, the TAS at a time delay of ca. 4 ps, which has an absorption band peaking at 525 nm (Fig. 1 and DAS *C* in Fig. 2a), compares satisfactorily with the spectrum calculated for the ^3^*π*_*s*_π* state, possibly with some contribution from the ^3^*n*_*s*_π* state (Fig. 3b).

**Figure 4:**
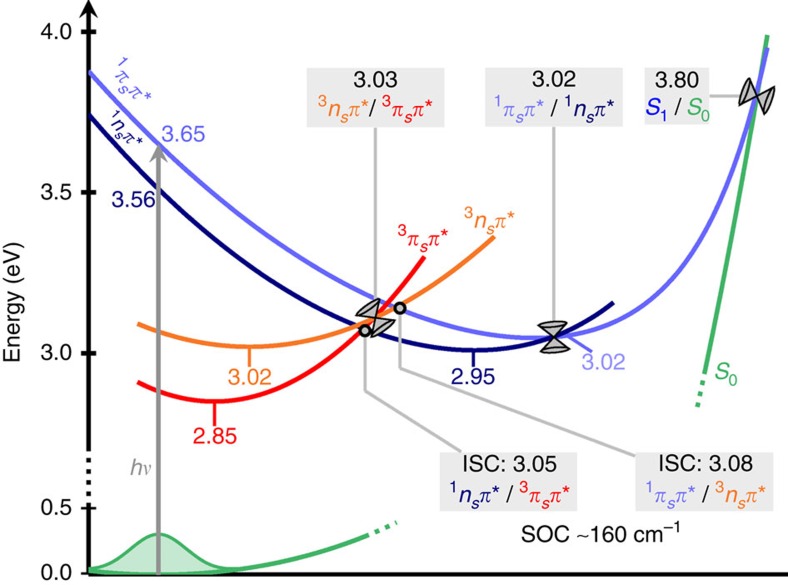
Schematic representation of possible relaxation pathways. All energies were computed at the MS-CASPT2 level of theory and are given in eV. Additional information can be found in Supplementary Note 2.

On the basis of static MS-CASPT2 calculations, the relaxation pathways sketched in Fig. 4 can be postulated (see Supplementary Table 3 for the corresponding data). After one-photon absorption (308 or 321 nm) the ^1^*π*_*s*_π* state at 3.65 eV is populated (*S*_2_ at the Franck–Condon geometry). A barrierless pathway leads to the ^1^*π*_*s*_π*/^1^*n*_*s*_π* CoIn at 3.02 eV, which connects to two different singlet state minima with ^1^*π*_*s*_π* (3.02 eV) or ^1^*n*_*s*_π* (2.95 eV) character. From these two minima, several relaxation pathways are plausible. Relaxation to the ground state involves overcoming energy barriers of at least 0.8 eV, making this process unlikely. Instead, two singlet-triplet (^1^*n*_*s*_π*/^3^*π*_*s*_π* and ^1^*π*_*s*_π*/^3^*n*_*s*_π*) minimum-energy crossing points (MECPs) located at 3.05 and at 3.08 eV, respectively, are accessible due to their small energy barriers and associated spin–orbit couplings of up to 170 cm^−1^. Energetically and geometrically close to these MECPs lies a CoIn between the ^3^*n*_*s*_π* and ^3^*π*_*s*_π* states, at an energy of 3.03 eV. Hence, there exists a three-state near-degeneracy region involving the lowest singlet and the two lowest triplet states. From the ^3^*n*_*s*_π*/^3^*π*_*s*_π* CoIn, the system can relax either to the ^3^*n*_*s*_π* minimum at 3.02 eV or to the ^3^*π*_*s*_π* minimum at 2.85 eV.

**Figure 5:**
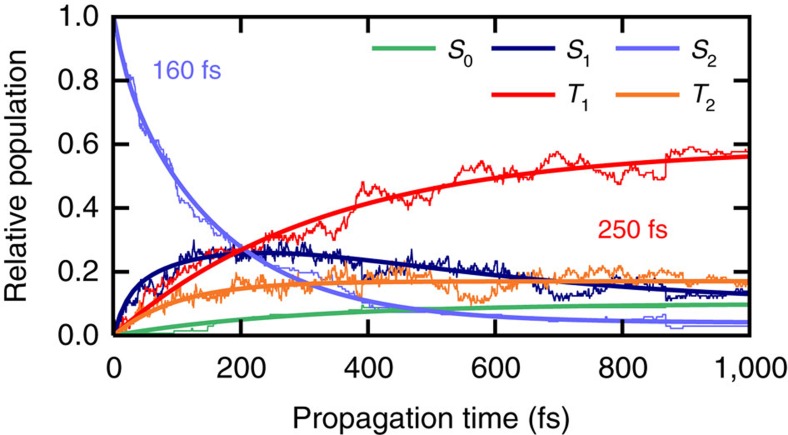
Excited-state populations from surface hopping simulations. Simulations were performed with the SHARC method coupled to the MRCIS electronic structure method. Thin lines represent the simulated electronic populations, thick lines show the fitted functions.

A time-resolved interpretation of the relaxation mechanism of 2tC is obtained by performing surface-hopping nonadiabatic simulations using the SHARC method (surface hopping including arbitrary couplings)^38^. Associated energies, gradients and relevant couplings are obtained at the multi-reference configuration interaction including single excitations (MRCIS) level of theory (see Supplementary Fig. 5 and Supplementary Note 2 for validation of the method). Figure 5 shows the excited-state populations based on 137 trajectories and the result of monoexponential fitting. The relaxation pathways in the dynamics simulations involve mainly four electronic states—labelled according to their energetic ordering at each geometry as *S*_1_, *S*_2_, *T*_1_ and *T*_2_—as foreseen by the quantum chemical calculations. These states encompass the ^1^*n*_*s*_π*, ^1^*π*_*s*_π*, ^3^*n*_*s*_π* and ^3^*π*_*s*_π* characters, but note that due to state crossing and mixing, no one-to-one correspondence between the *S*_*i*_/*T*_*i*_ labels and the characters exists beyond the Franck–Condon geometry.

After excitation, the population in the *S*_2_ state decays to the *S*_1_ state with a lifetime of 160 fs (Supplementary Table 4)—a value that might be slightly overestimated due to a small barrier to reach the *S*_2_/*S*_1_ crossing (0.13 eV), which is present with MRCIS but not with the more accurate MS-CASPT2 method (Fig. 4). The population of the *S*_1_ state rises to a maximum of about 30% during the first 200 fs due to population transfer from the *S*_2_ state. Afterwards, 2tC undergoes ISC to the triplet manifold with an average time constant of ∼250 fs. The population of the *S*_1_ state amounts to only 13% after 1 ps.

The most important ISC pathways predicted by the dynamics simulations are *S*_1_→*T*_2_ and *S*_1_→*T*_1_, with a minor contribution from *S*_2_→*T*_2_. The characters of the triplet states *T*_1_ and *T*_2_ are strongly mixed and cannot be assigned as strictly ^3^*π*_*s*_π* or ^3^*n*_*s*_π* during the dynamics simulations. It is precisely this mixed wavefunction character that leads to large spin–orbit couplings between *S*_1_ and both triplet states, explaining why both *S*_1_→*T*_1_ and *S*_1_→*T*_2_ transitions are possible, and why ISC is very efficient. According to the simulations, *T*_2_ is populated faster (in ∼100 fs) than *T*_1_ (in 330 fs) because the average *S*_1_→*T*_2_ energy gap is smaller than the *S*_1_→*T*_1_ gap. Note that the spin–orbit couplings vary in these regions but are on average ∼160 cm^−1^ between *S*_1_ and *T*_2_ as well as 50 cm^−1^ between *S*_1_ and *T*_1_. However, the *T*_1_ state receives most of the population within 1 ps due to subsequent *T*_2_→*T*_1_ internal conversion, leading to an average ISC lifetime of 250 fs. This value is in full agreement with the lifetime of 210±50 fs obtained experimentally. After 1 ps, 74% of the total population resides in either of the triplet states, and the remaining 16% of the population in the excited singlet manifold likely undergoes ISC at later times. A small fraction of the trajectories (10%) returned to the ground state because MRCIS underestimates the energy of the relevant *S*_1_/*S*_0_ CoIns, as compared with MS-CASPT2 (Supplementary Table 3). This ground-state repopulation should be regarded as an artefact of employing MRCIS in the dynamics simulations and we therefore estimate a >90% total ISC yield from the simulations.

From a structural point of view, there are no large out-of-plane deformations during the dynamics as anticipated from the similarity and quasi-planar nature of all excited-state minima (Supplementary Note 3). During the *S*_2_→*S*_1_ internal conversion, there is a bond inversion within the conjugated double bond system of the pyrimidine ring. As the trajectories relax to *S*_1_ and the triplet states, ring puckering increases, probably due to the increased available energy or due to a more flexible pyrimidine ring in these states. The triplet minima are structurally similar to the singlet minima and thus motion on the *T*_1_ and *T*_2_ PESs cannot be distinguished from motion on the *S*_1_ PES. In fact, the structural similarity is one of the factors leading to the efficient ISC observed in this work.

**Figure 6:**
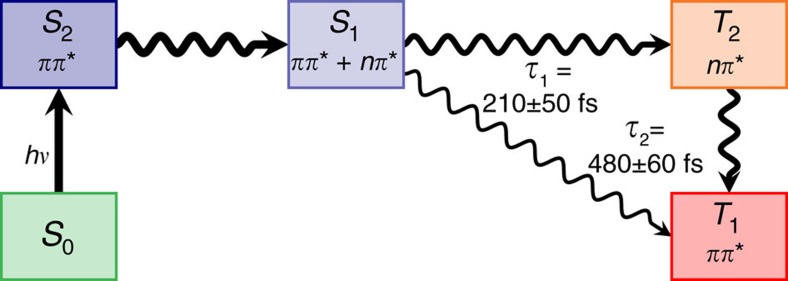
General relaxation mechanism of 2-thiocytosine. The scheme is based on the presented experimental and computational data. The *τ*_1_ and *τ*_2_ are the experimental time constants.

In summary, the experimental and theoretical results suggest that excitation of 2tC to the lowest-energy ^1^*π*_*s*_π* state (*S*_2_) leads to very efficient ISC to the ^3^*π*_*s*_π* (*T*_1_) state in three general steps, which are schematically depicted in Fig. 6. The first step is the relaxation from the vertically excited *S*_2_ (^1^*π*_*s*_π*) state to the *S*_1_ state through an *S*_2_/*S*_1_ CoIn. After transition from the *S*_2_ to the *S*_1_ state, the system populates the *S*_1_ PES, which in some regions of the configurational space has dominant ^1^*π*_*s*_π* character and in other regions ^1^*n*_*s*_π* character, according to MS-CASPT2 calculations. This is also consistent with the observation of a band assigned to the ^1^*π*_*s*_π* state in the TAS at short time delays. The second step is ultrafast ISC from the *S*_1_ state to the triplet manifold (*T*_2_ and *T*_1_ states). In the time-resolved experiments, this process leads to the appearance of an absorption band assigned to the ^3^*π*_*s*_π* state. This band begins to appear at approximately the same time as the ^1^*π*_*s*_π* absorption band, indicating that internal conversion in the singlet manifold and ISC occur on very similar time scales. The experimental time constant *τ*_1_ for this second step is 210±50 fs, in close agreement to the ISC lifetime of 250 fs observed theoretically. As the calculations show, the three-state near-degeneracy between *S*_1_, *T*_2_ and *T*_1_, together with the large spin–orbit matrix elements, are responsible for the ultrafast ISC lifetime of 2tC. Finally, the third step is internal conversion from the *T*_2_ to the *T*_1_ state and subsequent vibrational energy redistribution to populate the lowest-energy minimum of the *T*_1_ (^3^*π*_*s*_π*) state. The second experimental time constant of 480±60 fs is attributed to the latter step, together with residual ISC, as suggested by the decay of the UV transient absorption band. The eventual relaxation from the *T*_1_ state to the ground state appears to involve significant energetic barriers and does not play a role on the time scales considered in this work, in agreement with the long-lived nature of the *T*_1_ state of thiobases in solution^15^.

### Thiobases versus canonical bases

Having revealed the electronic relaxation mechanisms of 2tC, we are now in a favourable position to compare the structural and electronic features of the PESs in the thiobases and the canonical nucleobases, in order to address the intriguing question as to why they are so photophysically different. To this end, Table 1 collects the energetic parameters for cytosine (C), uracil (U) and guanine (G), and those of the corresponding thiobase analogues that have been investigated thus far—since the relative energies of the states minima and the *S*_1_/*S*_0_ surface crossings are strongly correlated to whether or not a nucleobase exhibits ultrafast relaxation to the ground state^39^.

The first conspicuous fact is that sulfur-substitution significantly lowers the energies of the singlet state minima. Both ^1^nπ* and ^1^ππ* state minima are stabilized by up to 1 eV for all three thiobases (2tC, 2tU, 6tG) in comparison with the corresponding canonical nucleobase. This stabilization is well-known in thiocarbonyl compounds^40^ and thiobases^15^,^30^. However, it is surprising that the energy of the most accessible (that is, lowest-energy) CoIn of each molecule stays approximately constant upon thionation (3.8/3.8 eV for C/2tC, 4.0/4.0 eV for U/2tU and 4.3/3.8 eV for G/6tG). As a consequence, canonical nucleobases exhibit efficient ground-state recovery after excitation, whereas the population of the thiobases rapidly decays to deep singlet state minima. The presence of one or more triplet states with energies similar to the singlet state minima and with large spin–orbit couplings facilitates subsequent ISC in the thiobases. While the energetic stabilizations can be correlated directly with the excited-state behaviour of the thiobases, it is not straightforward to rationalize why thionation lowers the energies of the states' minima so strongly, but has little impact on the energies of relevant CoIns.

We argue that a plausible explanation relates to the moiety of the molecule where the electronic excitation is localized. We focus on whether the excitation (that is, the excited electron or, more importantly, the hole left behind) is localized significantly on the (thio)carbonyl group or on the aromatic ring. States characterized by an excitation localized on the chalcogene atom are expected to stabilize considerably upon thionation because it takes less energy to excite an electron from an *n* or a *π* orbital of a sulfur atom into the π* system than from an *n* or *π* orbital of an oxygen atom (compare the lower ionization potential of S versus O)^41^. Conversely, if the electronic excitation is localized primarily on the pyrimidine ring (that is, electronic excitation from pyrimidine *π* to pyrimidine π*) no stabilization is expected upon thionation. Using the TheoDORE program^42^, the electronic wave functions were analysed to assign the localization of the relevant *S*_0_→*S*_1_ excitations. The results are shown in Table 1 (the corresponding difference densities of the excitations can be found in Supplementary Fig. 6, the numeric data in Supplementary Table 5). Remarkably, the stabilization of the excited-state minima of the thiobases can now be easily explained. Since the nπ* states are always localized on the carbonyl/thiocarbonyl group, the nπ* state minima are stabilized strongly upon thionation by ∼1 eV. Similar arguments explain why the ππ* states stabilize, but to lesser extent because they are at least partially delocalized over the aromatic rings.

In order to rationalize the energy shifts of the CoIns upon thionation, we consider C and 2tC as the initial example. Several different *S*_1_/*S*_0_ CoIns have been reported for C (Table 1)^43^,^44^,^45^,^46^,^47^,^48^,^49^. These CoIns have different electronic character and hence we expect different localization of the excitations. The excitation within the semiplanar CoIn is localized on the chalcogen atom and thus stabilized in 2tC. However, the ethylenic CoIn (which is the lowest-energy CoIn of C, and thus primarily responsible for its ultrafast relaxation) is not stabilized because its excitation is centred on the pyrimidine ring. The amino-out-of-plane (oop-NH_2_) CoIn is stabilized slightly (by 0.3 eV). Although the semiplanar CoIn is also stabilized by 1.4 eV in 2tC and becomes the lowest-energy CoIn, it is still too high in energy compared with the ^1^nπ* minimum, precluding ultrafast internal conversion to the ground state and favouring instead rapid decay to the ^1^nπ* minimum and subsequent ISC to the triplet manifold.

Satisfactorily, a similar rationale applies to the U/2tU pair. According to calculations, U relaxes primarily through the ethylenic CoIn^50^,^51^,^52^,^53^, with a twist at the carbon-carbon double bond, whereas the oxygen-out-of-plane (oop-O) CoIn^50^,^52^ is much higher in energy. In 2tU, on the other hand, the oop-S CoIn is stabilized, whereas the ethylenic CoIn is much higher in energy^26^,^54^. The stabilization of the oop-S CoIn is, however, not sufficient enough to provide a fast relaxation channel to the ground state^54^. To our knowledge, there are no calculations on 2-thiothymine, but we expect that similar arguments hold for the thymine/2-thiothymine pair, because the methyl group is not expected to affect the electronic structure significantly.

The CoIns in the G/6tG pair also follow the discussed trend: the oop-S CoIn is stabilized more than the oop-NH_2_ CoIn—although the effect is less pronounced than in the pyrimidine bases. Ground-state relaxation from the ^1^ππ* minimum in 6tG would be energetically possible, but the shape of the PES favours the population of the ^1^nπ* minimum instead, from where all possible deactivation pathways to *S*_0_ involve very large energy barriers.

From the above analysis, it can be proposed that thionation of the nucleobases stabilizes all states in which the electronic excitation is primarily localized on the sulfur atom (for example, *n*_*s*_π*, *π*_*s*_π*), whereas the other states (*n*_*N*_π*, *π*_ring_π*), and their corresponding CoIns, are not stabilized to the same extent. This leads to the conclusion that the intrinsic relaxation pathways in the canonical nucleobases—which involve extensive ring puckering but no extensive motion of the carbonyl group—are blocked in the thiobases because they are too high in energy relative to the considerably stabilized excited-state minima. Although there exist *S*_0_/*S*_1_ CoIns involving sulfur-localization that are also stabilized^25^,^29^,^54^, they are too high in energy with respect to the excited-state minima and do not play a role in the deactivation.

Finally, localization of the excitation on the sulfur atomic orbitals also enhances spin–orbit coupling. This is because the spin–orbit operator is of short range nature^55^ and depends on the atomic number, thus making this coupling largest if the excitations are localized on the heaviest atom. Further enhancement of population transfer from the singlet to the triplet manifold is due to the smaller singlet-triplet energy gaps in the thiobases compared with the canonical nucleobases because heavy atom substitution often leads to an increased density of states.

## Discussion

Using transient absorption spectroscopy and theoretical calculations, we demonstrated that photoexcited 2-thiocytosine populates the triplet manifold on a femtosecond timescale and with close-to-unity triplet yield. Its relaxation after excitation proceeds in three general steps, which partially overlap: (i) barrierless deactivation from the *S*_2_ to the *S*_1_ state; (ii) ISC to the triplet manifold with a time constant of 210±50 fs, enhanced by the presence of a three-state near-degeneracy region involving the *S*_1_, *T*_2_ and *T*_1_ states; and (iii) internal conversion to the *T*_1_ state. The latter step, together with some residual ISC is responsible for a time constant of 480±60 fs. More importantly, we have rationalized and generalized the influence of thionation on the excited-state dynamics of the canonical nucleobases. While it is generally recognized that thionation leads to a strong decrease in excited-state energies, here we show that the extent of this stabilization differs significantly in different parts of the PESs. Furthermore, we have shown that the stabilization depends sensitively on the electronic wavefunction character and favours wave functions where the excitation is localized on the sulfur atom. Hence, excited-state minima (for example, ^1^*n*_*s*_π*) and conical intersections involving the thiocarbonyl group are stabilized upon thionation, whereas the conical intersections intrinsic to the pyrimidine skeleton are not. This observation explains satisfactorily the remarkably different behaviour of thiobases compared with their parent nucleobases and provides design criteria for the synthesis of prospective photochemotherapeutic drugs.

## Methods

### Materials

2-Thiocytosine (97%, Sigma-Aldrich), sodium dihydrogenphosphate (99.0%, Sigma-Aldrich), and sodium hydrogenphosphate (99.0%, Sigma-Aldrich) were used as received. PBS solutions were prepared using 0.15 g of sodium dihydrogenphosphate and 0.27 g of sodium hydrogenphosphate dissolved in 200 ml of ultrapure water to give pH 7.4 aqueous phosphate buffer with a total phosphate concentration of 16 mM.

### Femtosecond transient absorption spectroscopy

Femtosecond broadband transient absorption spectroscopy was performed using a Libra-HE pulsed laser system (Coherent, Inc.), which produces 800 nm, 100 fs pulses with a total output power of 4 W. A small fraction of this fundamental beam (<2 mW) was split off and focused into a continuously moving 2 mm CaF_2_ crystal to generate the white light continuum probe pulses (320–710 nm). The remainder of the fundamental beam was directed through an optical parametric amplifier (TOPAS-800, Light Conversion, Ltd.) to generate the excitation (that is, pump) pulses at either 308 or 321 nm. All other wavelengths were removed from the excitation beam using a reflective wavelength filter and a Glan-Taylor polarizer.

The spectrometer (Helios, Ultrafast Systems, LLC.) uses an optical delay line to delay the probe pulses in time with respect to the pump. The pump power was attenuated to 1 μJ at the sample and its polarization was randomized using a depolarizing plate in order to prevent contribution of rotational effects to the dynamics. At each delay time the spectrum of the probe is collected with and without pumping the sample in order to produce the difference spectra (ΔA).

2tC solutions were prepared at concentrations of 5–8 mM in pH 7.4 aqueous PBS. All transient absorption experiments were performed in 2 mm optical path length quartz cuvettes (Starna Cells, Inc.). Sample solutions were stirred continuously throughout data collection and replaced with fresh sample, from the same stock, every 5–7 scans so that all scans had no more than 5% degradation as determined by the steady-state absorption. A home-made LabView program (National Instruments, Inc.) was used to correct all transient absorption data for group velocity dispersion of the white light probe^56^. Global and target analysis of the data were performed using a sequential kinetic model^22^, convoluted with the instrument response function fixed at 200 fs, as determined by the coherence signal of neat methanol^12^.

The sequential kinetic model included two lifetimes:


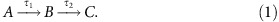


The spectrum of the third component *C* can be considered as an offset and represents the transient species that has not decayed during the first 20 ps investigated in this work.

The results of five separate experiments, three exciting at 308 nm and two exciting at 321 nm, were analysed. The target and global fitting analysis was performed independently for each of the five experiments in order to estimate the uncertainty of the lifetimes. Up to 91 kinetic traces from the broadband transient absorption data were used in this analysis. The uncertainties are reported as twice the standard deviation. The DAS are extracted from the global fit of the data and correspond to the wavelength-dependent amplitudes for each of the lifetime components in the kinetic traces.

### Stationary quantum chemistry

According to Kasha's rule, the relaxation from *S*_4_ via *S*_3_ to *S*_2_ should be ultrafast. This is in agreement with the experimental fs-TAS results, which showed that excitation at 321 nm (mainly to *S*_2_) and excitation at 308 nm (which also excites *S*_4_) induce the same excited-state dynamics. Hence, the computational investigations focused on the relaxation dynamics starting from *S*_2_.

Minima of the PESs were first optimized at the CASSCF(14,10)/ANO-S-VDZP level of theory, whereas for the optimization of CoIns and MECPs CASSCF(14,10)/6-31G* was used. Using CASSCF(14,10)/ANO-S-VDZP, all minima and crossing points were connected with minimum-energy paths to check for the absence of energetic barriers. If a minimum-energy path successfully finishes from a surface crossing to a minimum, it implies that there are no energetic barriers in between. The minima, CoIns and MECPs were then reoptimized at the MS-CASPT2/ANO-L-VTZP level of theory using the methodologies described in refs. 57, 58 and the ORCA optimizer^59^. For all MS-CASPT2 calculations, an imaginary level shift of 0.3 a.u. and the default IPEA shift of 0.25 a.u. has been used. All calculations were conducted in the gas phase and using MOLCAS 7.8 (ref. 60), except for the CASSCF optimizations of the CoIns and MECPs, where MOLPRO 2009 (ref. 61) was used.

The levels of theory used in the various calculations, including the numbers of averaged states, are also compiled in Supplementary Table 6. The active space or reference space orbital composition is presented in Supplementary Fig. 7. All calculations were performed with the amino-thion tautomer of 2-thiocytosine, which is justified in Supplementary Fig. 8 and Supplementary Table 7. All optimized geometries are reported in Supplementary Note 3.

While MS-CASPT2 is, in many cases, an excellent method to describe excited states, it cannot be yet routinely employed in dynamics simulations, due to the lack of efficient implementations of analytical MS-CASPT2 nuclear gradients. Hence, all stationary quantum chemistry calculations were repeated with MRCIS (as specified in ‘nonadiabatic dynamics simulations') to scrutinize the accuracy of the MRCIS PESs employed in the dynamics simulations versus the MS-CASPT2 ones. See Supplementary Fig. 5, Supplementary Table 3 and Supplementary Note 2 for further discussions.

### Nonadiabatic dynamics simulations

The on-the-fly electronic structure level of theory for the nonadiabatic dynamics simulations was MRCIS with a reference space of CAS(6,5) type. The orbitals were taken from previous CASSCF(10,8)/cc-pVDZ calculations (see Supplementary Fig. 7 for the orbitals used), with state-averaging over four singlet and two triplet states (note that the number of states in the MRCIS calculations does not need to equal the number of states in the SA-CASSCF calculations). The CAS(6,5) has been created removing the least occupied π*-orbital and the most occupied two *π*-orbitals from the CAS(10,8), and therefore the reduced reference space still contains all orbitals involved in the primary excitations of all the relevant states. Inner shells were kept frozen in the MRCIS stage of the calculations. All MRCIS calculations were conducted with COLUMBUS 7 (ref. 62), with integrals from MOLCAS^60^, which also provided scalar-relativistic one-electron integrals according to the Douglas–Kroll–Hess method and the spin–orbit integrals using atomic mean-field integrals 55.

The surface hopping code SHARC was employed^38^. Surface hopping is an extension of classical molecular dynamics to include nonadiabatic transitions between excited states, and SHARC in turn is an extension of surface hopping to include other types of couplings beyond nonadiabatic ones. In the present study, the additional couplings were the spin–orbit couplings among the states *S*_0_ to *S*_3_ and *T*_1_ to *T*_2_. Within SHARC, these states are mixed to yield ‘diagonal' states, on whose PESs the dynamics is carried out; this procedure includes calculating the gradients of the diagonal states from the spin-free gradients as well as propagating the electronic wavefunction using the spin–orbit Hamiltonian and the nonadiabatic couplings of the spin-free states. SHARC is available free of charge from http://sharc-md.org, and the development version from the authors upon request.

Initial conditions for the dynamics simulations were obtained by sampling from a quantum-harmonic Wigner distribution around the ground state minimum, based on a frequency calculation performed with the MRCIS method. 4,000 geometries were sampled from the distribution and for each geometry the vertical excitation energies and oscillator strengths were calculated, which were used to simulate an absorption spectrum (Supplementary Fig. 2) and to select the initial electronic state for the dynamics.

One hundred thirty seven trajectories were propagated for a total simulation time of up to 1 ps. The time step for the nuclear motion was 0.5 fs, while the electronic Schrödinger equation was integrated with a time step of 0.02 fs using the local diabatization methodology^63^, which employs wavefunction overlaps^64^ to provide the information about the nonadiabatic couplings. An energy-based decoherence correction^65^ was applied to the electronic states in the diagonal basis. As stated above, the simulations were carried out on spin-adiabatic potentials, obtained by diagonalization of the spin–orbit Hamiltonian including states *S*_0_ to *S*_3_ and *T*_1_ to *T*_2_. However, for the analysis of the results the state populations were transformed back into the spin-free basis, since this allows for easier interpretation of the results. Note that the energies of *S*_4_, *T*_3_ and *T*_4_ (but no gradients, spin–orbit couplings or wavefunction overlaps) were also calculated at each time step in order to scrutinize whether neglecting these states is justified. Furthermore, since the *S*_3_ population decays within only 20 fs to the *S*_2_ state, for all analysis and discussion purposes the *S*_3_ population was merged into the *S*_2_ population.

### Wave function localization analysis

The calculations were performed using optimized geometries reported in the literature for cytosine^48^, uracil^43^,^52^, guanine^66^, 2-thiouracil^54^ and 6-thioguanine^25^,^29^, whereas for 2-thiocytosine the geometries reported here were used. For each molecule, the nπ* and ππ* excited-state minima and relevant CoIns were included. At each geometry, a CASSCF/ANO-L calculation was performed, averaging over 4 singlet states. For cytosine, uracil, 2-thiocytosine, and 2-thiouracil, a CAS(14,10) was used, whereas for guanine and 6-thioguanine a CAS(18,13) was employed. These calculations were performed with MOLCAS^60^. The one-electron difference density matrices between the ground state and the first excited state were then analysed using the TheoDORE program^42^ in terms of the Mulliken detachment populations in order to arrive at the classification in Table 1. The Mulliken populations are given in Supplementary Table 5.

### Data availability

All data is available from the authors upon request.

## Additional information

**How to cite this article:** Mai, S. *et al*. The origin of efficient triplet state population in sulfur-substituted nucleobases. *Nat. Commun.* **7,** 13077 doi: 10.1038/ncomms13077 (2016).

## Supplementary Material

Supplementary Information Supplementary Figures 1-8, Supplementary Tables 1-7, Supplementary Notes 1-3 and Supplementary References

## Figures and Tables

**Table 1:** Relative energies of stationary points and crossings for 2tC, 2tU, 6tG, and their parent nucleobases.

Critical Point	Localization on O	ΔE	Localization on S	ΔE
	**C (ref. 43)**		**2tC^ (this work)^**	
^1^nπ* min	Yes	4.2	Yes	3.0 ↓
^1^ππ* min	Partially	4.0	Partially	3.0 ↓
*S*_0_/*S*_1_ semiplanar	Yes	5.2	Yes	3.8 ↓
*S*_0_/*S*_1_ oop-NH_2_	No	4.1	Partially	3.8 ↓
*S*_0_/*S*_1_ ethylenic	No	3.8	—*	High ↑
Behaviour	Relaxation (2–3 ps (refs 67, 68))		ISC (210±50 fs)	
	**U (ref. 50)**		**2tU (ref. 54)**	
^1^nπ* min	Yes	4.1	Yes	3.3 ↓
^1^ππ* min	No	4.5	Yes	3.8 ↓
*S*_0_/*S*_1_ oop-Y (Y=O/S)	Yes	5.8	Yes	4.0 ↓
*S*_0_/*S*_1_ ethylenic	No	4.0	—*	High ↑
Behaviour	Relaxation (2.5 ps (refs 68, 69))		ISC (360±30 fs (ref. 12))	
	**G (ref. 66)**		**6tG (ref. 25)**	
				
^1^nπ* min	Yes	4.3	Yes	3.1 ↓
^1^ππ* min	Partially	4.1	Partially	3.8 ↓
*S*_0_/*S*_1_ oop-Y (Y=O/S)	No	4.4	Yes	3.8 ↓
*S*_0_/*S*_1_ oop-NH_2_	No	4.3	No	3.9 ↓
Behaviour	Relaxation (360 fs (ref. 70))		ISC (310±50 fs (ref. 20))	

All energies (Δ*E*) are given in eV above the ground state minimum of the respective molecule. The classification whether the *S*_0_→*S*_1_ excitation is localized on the chalcogen atom (Y=O/S) is based on the density plots presented in Supplementary Fig. 6 and Supplementary Table 5. These plots were calculated at the CASSCF(14,10) level of theory (CASSCF(18,13) for G and 6tG).

^*^Not reported in the literature, but presumed to have no localization on S in analogy to the equivalent CoIn in the parent nucleobase.
