# Supplementary material for: The origin of efficient triplet state population in sulfur-substituted nucleobases
Source: Nat Commun. 2016 Oct 5;7:13077. doi: 10.1038/ncomms13077 (PMC5059480; doi:10.1038/ncomms13077)
Supplement: Supplementary Information — Supplementary Figures 1-8, Supplementary Tables 1-7, Supplementary Notes 1-3 and Supplementary References [file ncomms13077-s1.pdf]

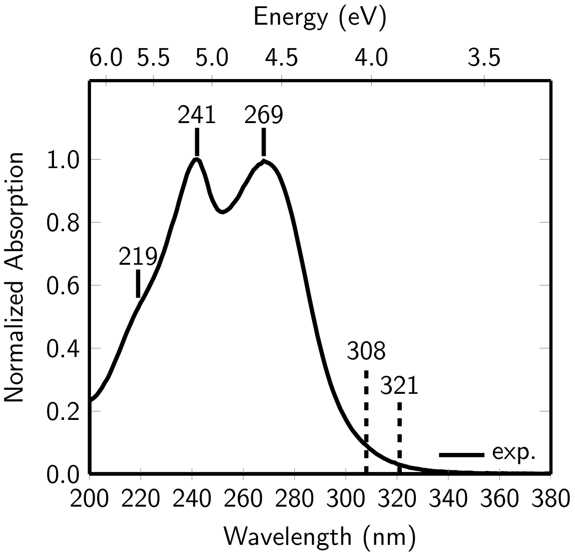

Supplementary Figure 1: Steady-state absorption spectrum. The black line corresponds to the absorption spectrum in phosphate buffer saline solution, whereas the dashed lines indicate the excitation wavelengths (308 and 321 nm) used in the femtosecond transient absorption experiments. The solid ticks mark the two absorption maxima of nearly identical intensities at 241 and 269 nm and the shoulder at 219 nm. Residual absorption extending to about 350 nm is also apparent in the spectrum. The absorption spectrum was recorded using a Cary 100 Bio UV-Vis Spectrophotometer (Varian) in 1 cm optical path quartz cells (Starna Cells, Inc.), and was corrected for the background signal of the solvent.

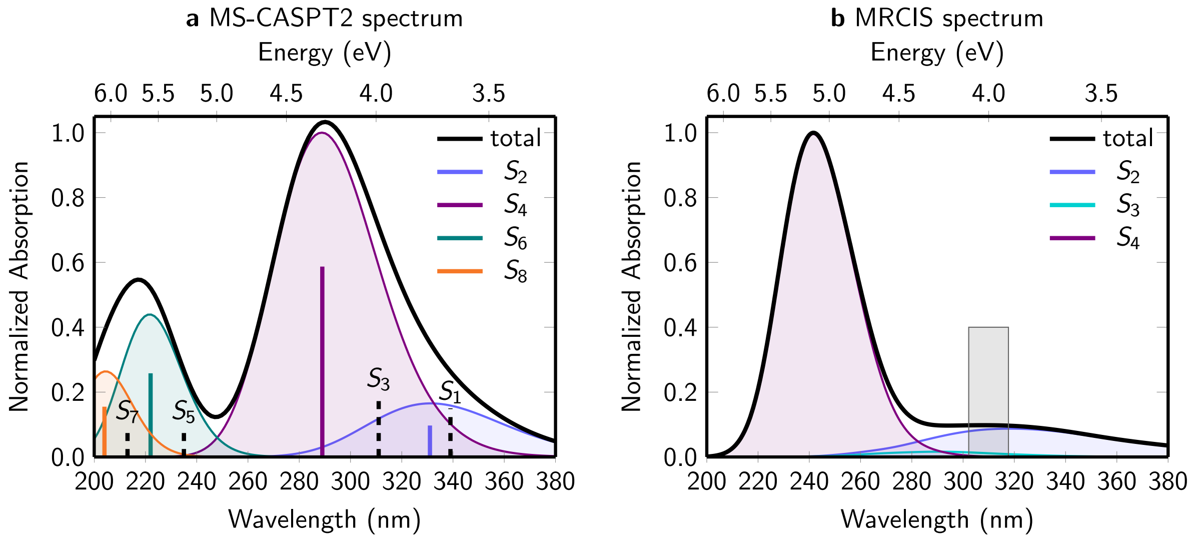

Supplementary Figure 2: Computed ground state absorption spectra. The spectrum in panel **(a)** was computed as the sum over Gaussians (FWHM of 0.7 eV) centered at the vertical excitation energy and with height proportional to the oscillator strength, based on the MS-CASPT2 results from Supplementary Table 1. Dashed lines indicate the position of the dark $n\pi^*$ states. The spectrum in panel **(b)** was simulated using the line broadening method,[1] based on 4000 geometries sampled from the Wigner distribution of the ground state harmonic vibrational wave function. At each of the geometries a single point calculation of 4 excited states was performed with the MRCIS method employed in Supplementary Table 1. The total spectrum was computed as a sum of Gaussians (FWHM of only 0.3 eV, since the distribution of geometries already accounts for most of the broadening effect) including all 16000 transitions (4 excited states $\times$ 4000 geometries). The grey box denotes the energy window from where initial conditions for the dynamics were selected. More discussion can be found in Supplementary Note 1.

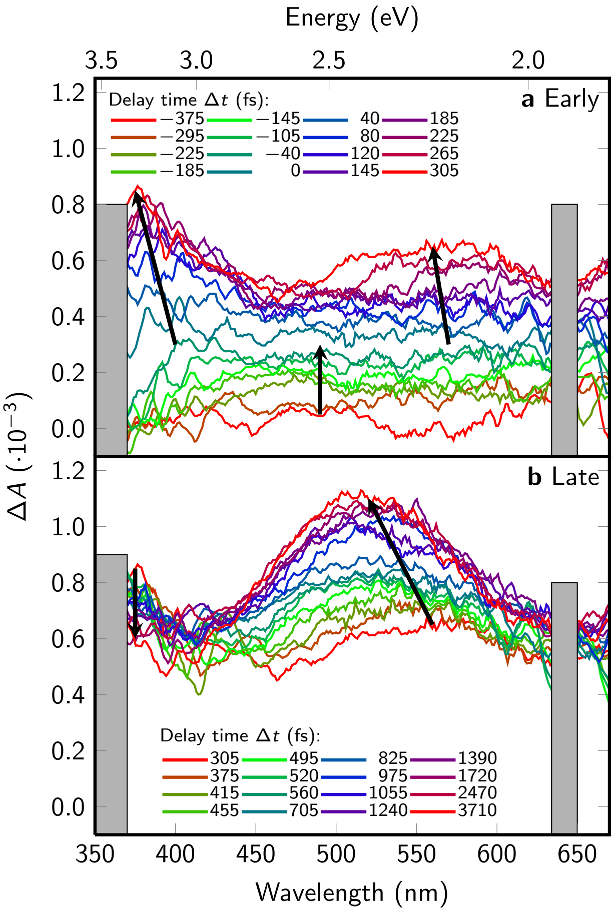

Supplementary Figure 3: Transient absorption spectra after excitation with 321 nm radiation. Panel **(a)** presents the transient spectra at early delay times (-375 to 305 fs), and panel **(b)** the ones at later delay times (305 fs to 3710 fs). Arrows indicate the grow/decay of the absorption bands with time. The grey boxes block the overtone band of the excitation light (ca. 642 nm) and the stimulated Raman emission due to the overlap of the pump and probe pulses (ca. 360 nm). All spectra were recorded in aqueous buffer solution. Although the data is noisier than the TAS obtained with 308 nm excitation (Fig. 1 in the main manuscript), the two TAS agree well with each other. In the 321 nm TAS, initially (at negative time delays) a constant rise across the whole spectral range is observed. Around 0 fs time delay two bands begin to appear, one with a maximum below 400 nm and the second, broader band, at wavelengths around 550 nm. At a time delay of 305 fs, the 400 nm band reaches its maximum intensity at approximately 375 nm, whereas the band around 550 nm continues to grow. Only at a time delay of about 3.7 ps does the latter band reach its maximum around 525 nm.

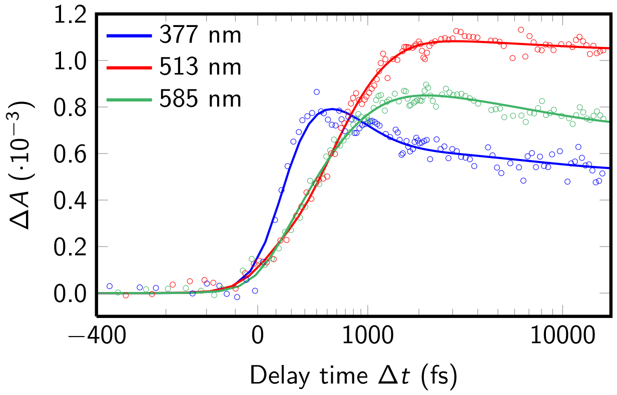

Supplementary Figure 4: Representative kinetic traces for excitation at 321 nm. The solid lines present the fitted functions from the target and global analysis. Just as with the 308 nm data, the 321 nm data requires a sequential kinetic model with two lifetimes for an adequate fit of the data. The lifetimes obtained from fitting the 321 nm data agree within error with the lifetimes from the 308 nm data. Reported in the main manuscript are the average lifetimes including both the 321 nm and 308 nm data: τ_1=210 ± 50 fs and τ_2=480 ± 60 fs.

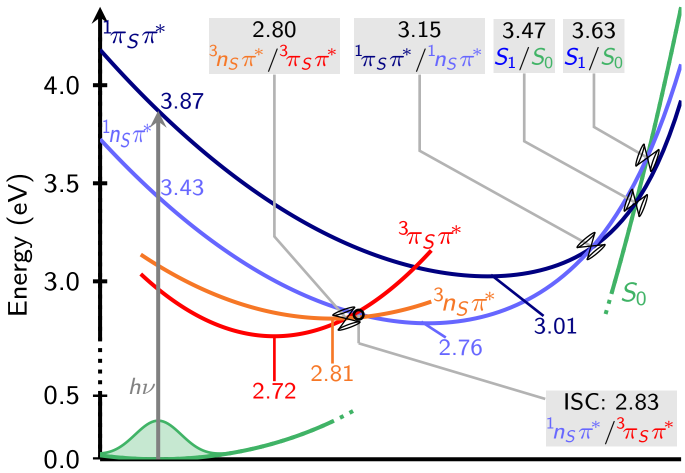

Supplementary Figure 5: Relaxation mechanism scheme according to MRCIS. All energies are given in eV. See Supplementary Table 6 for details on the level of theory. The discussion of this figure is given in Supplementary Note 2.

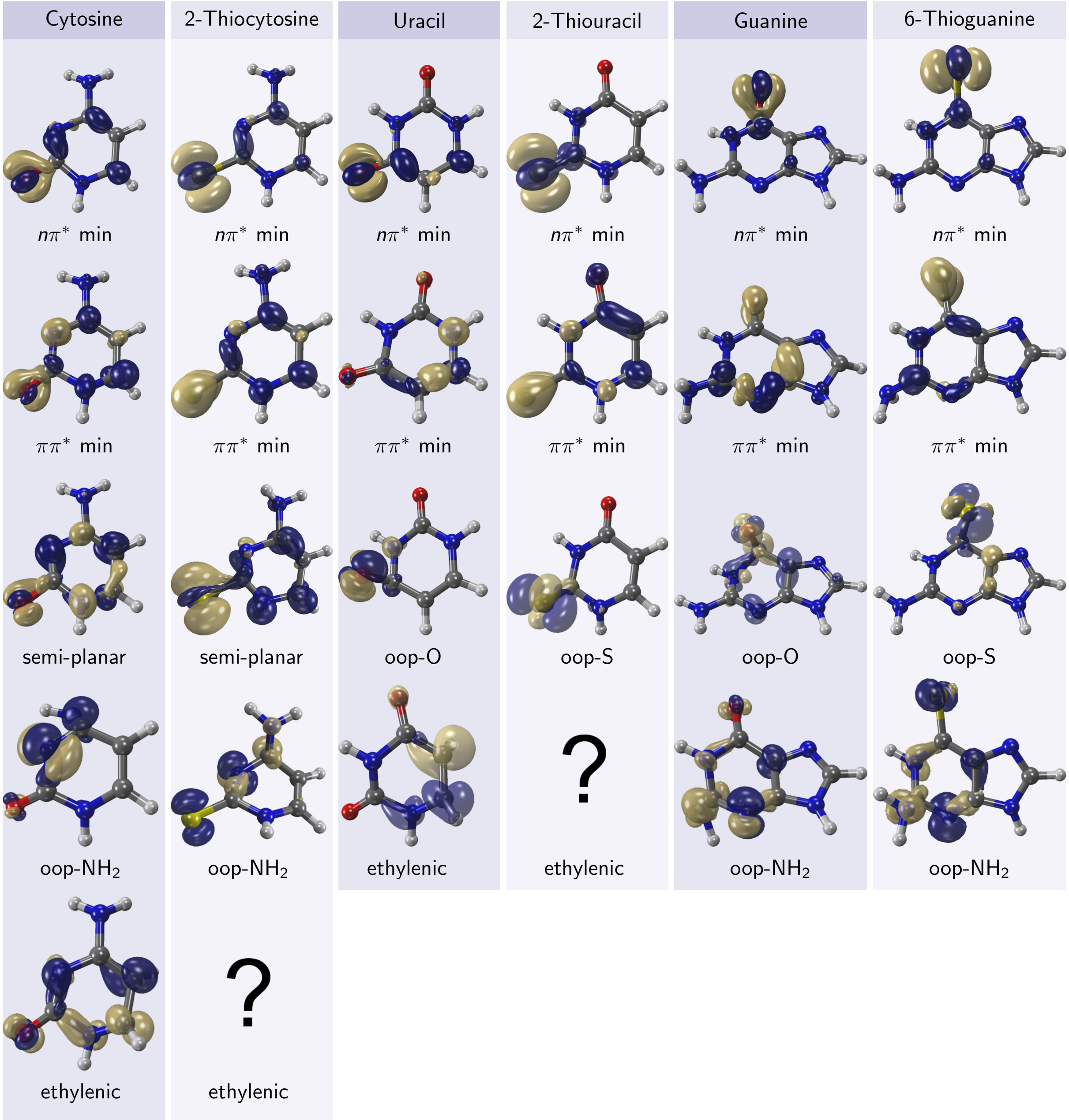

Supplementary Figure 6: Difference density plots showing the localization of the electronic excitation. In the plots, gold color denotes the detachment density (i.e., where the electron was excited from) and blue color denotes the attachment density (where the electron was excited to). In all cases, the $S_0 \rightarrow S_1$ transition has been plotted, as calculated with CASSCF/ANO-L. Question marks denote the "ethylenic" CoIns of 2-thiocytosine and 2-thiouracil, which are not reported in the literature, although for C and U the equivalent CoIns play an important role. For cytosine, three CoIns are shown, and it can be observed that the semi-planar CoIn shows a rather delocalized excitation, while the attachment and detachment densities are localized on the C=N double bond for the oop-NH_2 CoIn and on the C=C double bond for the ethylenic CoIn. On the contrary, in 2-thiocytosine, the semi-planar CoIn shows localization of the detachment density to the sulfur atom, which lowers the energy of the CoIn compared to the equivalent CoIn in cytosine. The oop-NH_2 CoIn of 2-thiocytosine exhibits only a smaller degree of localization on the sulfur atom. In general, the CoIns displayed in the third row show the highest degree of difference density localization on oxygen/sulfur, while the fourth and fifth rows show CoIns with difference density localized on the pyrimidine/purine backbone.

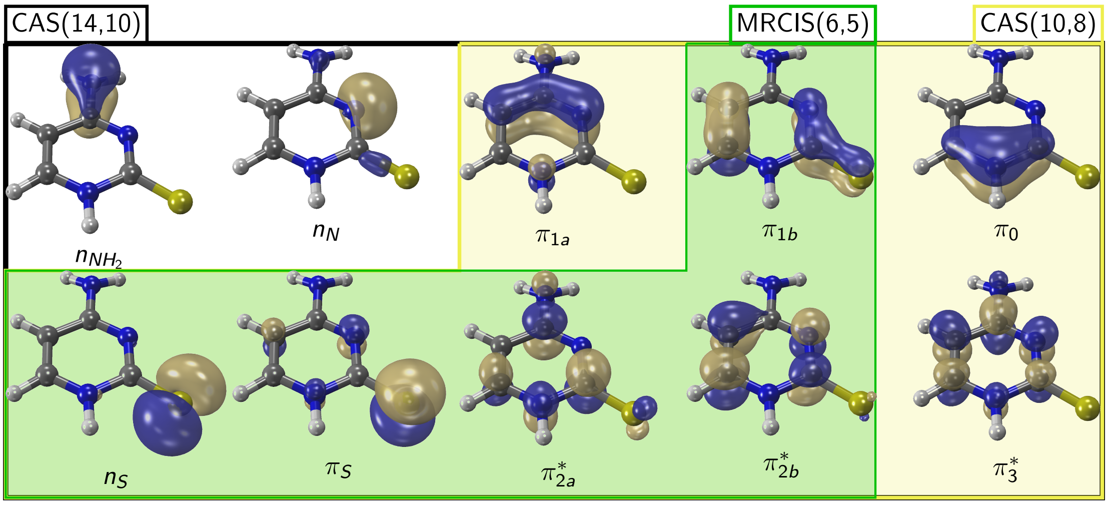

Supplementary Figure 7: Active space/reference space orbital composition for all computations. The levels of theory are CASSCF(14,10) and CASSCF(10,8)+MRCIS(6,5). See Supplementary Table 6 for an overview of the levels of theory used to produce each result.

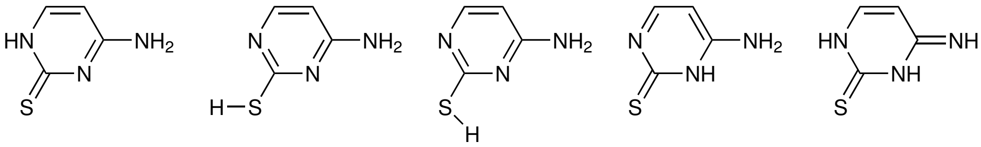

Supplementary Figure 8: Chemical structures of tautomers of 2-thiocytosine. These tautomers are reported to be the most stable ones for 2-thiocytosine.[2] From left to right, these are the amino-thion-N_1H (TC1), amino-thiol (with rotamers TC2a and TC2b), amino-thion-N_3H (TC3), and imino-thion (TC4) forms. The absolute and relative energies of these tautomers in gas phase and aqueous solution are given in Supplementary Table 7. In the gas phase, TC2 is the most stable tautomer, with an almost 1:1 ratio between the rotamers TC2a and TC2b. The other tautomers are too high in energy, and can be expected to be absent in vacuo. In solution, the very polar TC1 and TC3 tautomers are strongly stabilized and TC1 becomes the most stable tautomer. Based on the energies obtained, we expect that in solution the only tautomer present is TC1. This finding agrees with several other quantum-chemical calculations reported in the literature,[3–8] including one study which included explicit solute-solvent interactions,[2] and experimental studies.[9,10] Based on these results we focus on the TC1 tautomer. This tautomer is present in aqueous solution (the solvent used in the experiments) and is the biologically most relevant one (since TC1 forms 2-thiocytidine).

Supplementary Table 1: Vertical excitation energies, oscillator strengths and characters of the lowest excited states. The employed levels of theory for the calculations are MS-CASPT2(14,10)/ANO-RCC-VQZP//RI-MP2/cc-pVQZ and CASSCF(10,8)+MRCIS(6,5)/cc-pVDZ. All calculations were performed in vacuo. Both electronic structure methods predict that the four lowest excited singlet states are centered on the thiocarbonyl group—two states with $n_S\pi^*$ character (S_1 and S_3) and two states with $\pi_S\pi^*$ character (S_2 and S_4). Higher-lying states originate from excitation from the nitrogen lone pair ($n_N\pi^*$) and from excitation within the pyrimidine π system ($\pi\pi^*$) according to MS-CASPT2. Based on the calculated oscillator strengths, the most intense transitions are the $\pi_S\pi^*$ (S_4) and $\pi\pi^*$ (S_6) states, with energies of 4.29 and 5.59 eV, respectively. The lowest $\pi_S\pi^*$ state (S_2), at 3.74 eV, is an order of magnitude weaker than the S_4 and S_6 states. The $n_S\pi^*$ and $n_N\pi^*$ states are also dark according to the calculations. The four lowest triplet states are also centered on the thiocarbonyl group (two $n_S\pi^*$ and two $\pi_S\pi^*$ states) but with a different state ordering than the singlet states. The higher triplet states originate from either a pyrimidine $\pi\pi^*$ transition or an $n_N\pi^*$ transition, based on the MS-CASPT2 calculations.

State	MS-CASPT2			MRCIS		
	E (eV)	f_{osc}	Char.	E (eV)	f_{osc}	Char.
S_0	0.00	—	GS	0.00	—	GS
S_1	3.65	0.001	$n_S\pi^*$	3.43	0.000	$n_S\pi^*$
S_2	3.74	0.097	$\pi_S\pi^*$	3.87	0.047	$\pi_S\pi^*$
S_3	3.99	0.001	$n_S\pi^*$	4.03	0.000	$n_S\pi^*$
S_4	4.29	0.587	$\pi_S\pi^*$	5.20	0.465	$\pi_S\pi^*$
S_5	5.26	0.003	$n_N\pi^*$			
S_6	5.59	0.258	$\pi\pi^*$			
S_7	5.83	0.005	$n_N\pi^*$			
S_8	6.06	0.155	$\pi\pi^*$			
T_1	3.37	—	$\pi_S\pi^*$	3.36	—	$n_S\pi^*$
T_2	3.47	—	$\pi_S\pi^*$	3.40	—	$\pi_S\pi^*$
T_3	3.64	—	$n_S\pi^*$	3.74	—	$\pi_S\pi^*$
T_4	3.98	—	$n_S\pi^*$	4.01	—	$n_S\pi^*$
T_5	4.62	—	$\pi\pi^*$			
T_6	5.14	—	$n_N\pi^*$			
T_7	5.73	—	$\pi\pi^*$			
T_8	6.32	—	$n_N\pi^*$			

Supplementary Table 2: Vertical excitation energies and oscillator strengths for excited-state absorption. These results were computed at the MS-CASPT2/CASSCF(14,10)/ANO-L//CASSCF(14,10)/ANO-S level of theory. Only the relevant bright absorptions for each minimum are given (the brightest ones are given in bold). This data was used to simulate the theoretical TAS presented in Fig. 3 in the main manuscript.

From	To	ΔE (eV)	ΔE (nm)	f_{osc}
$^1\pi_S\pi^*$ (S_2)	$\mathbf{S_6}$	**3.41**	**363**	**0.0646**
$^1n_S\pi^*$ (S_1)	$\mathbf{S_5}$	**2.15**	**576**	**0.0043**
	S_6	3.72	333	0.0023
$^3n_S\pi^*$ (T_2)	$\mathbf{T_6}$	**2.16**	**574**	**0.0018**
	T_8	3.73	333	0.0015
$^3\pi_S\pi^*$ (T_1)	$\mathbf{T_6}$	**2.27**	**546**	**0.0319**
	$\mathbf{T_7}$	**2.81**	**441**	**0.0390**
	T_8	3.87	320	0.0019

Supplementary Table 3: Energies of important excited-state minima and crossing points. Energies are given in eV relative to the S_0 minimum energy at the respective level of theory. Energy gaps are given in eV. Bold numbers indicate the states involved in the three-state near-degeneracy region, as mentioned in the main text. Wave function character is given where possible, otherwise adiabatic state ordering is given. These results were computed at the MS-CASPT2(14,10)//CASSCF(14,10)/ANO-L or CASSCF(10,8)+MRCIS(6,5)/cc-pVDZ level of theory. More discussion is given in Supplementary Note 2.

Geometry	MS-CASPT2		MRCIS	
	Energy	Gap	Energy	Gap
$^1n_S\pi^*$ at S_0 min	3.56	—	3.43	—
$^1\pi_S\pi^*$ at S_0 min	3.65	—	3.87	—
Min $^1n_S\pi^*$	**2.95**	—	**2.76**	—
Min $^1\pi_S\pi^*$	**3.02**	—	3.01	—
Min $^3\pi_S\pi^*$	2.85	—	**2.72**	—
Min $^3n_S\pi^*$	**3.02**	—	**2.81**	—
CoIn $^1n_S\pi^*/^1\pi_S\pi^*$	**3.02**	< 0.01	3.14	< 0.01
CoIn $^3\pi_S\pi^*/^3n_S\pi^*$	**3.03**	0.02	**2.80**	0.04
MECP $^1n_S\pi^*/^3\pi_S\pi^*$	**3.05** [a]	< 0.01	**2.83**	0.01
MECP $^1\pi_S\pi^*/^3n_S\pi^*$	**3.08** [b]	< 0.01	—	—
CoIn S_1/S_0 semiplanar 1	3.82	0.01	3.47	< 0.01
CoIn S_1/S_0 semiplanar 2	3.80	0.02	3.63	< 0.01
CoIn $^1\pi\pi^*/S_0$ oop-NH_2	3.80	< 0.01	3.68	< 0.01

[a] Spin-orbit coupling: 160 cm^{-1}.
[b] Spin-orbit coupling: 170 cm^{-1}.

Supplementary Table 4: Fitting parameters for the populations in the SHARC dynamics simulations. The ensemble populations (137 trajectories) of all states (see Fig. 5 in main manuscript) except S_1 were fitted with monoexponential functions of the form $f(x) = (A - B)e^{-\frac{t}{\tau}} + B$, where A and B are the populations at $t = 0$ and $t = \infty$, respectively, and τ is the monoexponential time constant. The population of S_1 was not fitted, since it it an intermediate state and its lifetime is mathematically correlated with the other lifetimes.

States	A (%)	B (%)	τ (fs)	Type
S_0	0	10	290	Rising
S_2	87	4	160	Decay
S_3	13	0	20	Decay
T_1	0	59	330	Rising
T_2	0	17	100	Rising
$T_1 + T_2$	0	74	250	Rising
S_1	— Remainder of the population —			

Supplementary Table 5: Mulliken detachment populations. This data describes how much electron density is removed from the oxygen/sulfur atom during the respective excitation. Values smaller than 0.2 are denoted "no" in Table 1 in the main manuscript, values between 0.2 and 0.5 as "partially", and values above as "yes". The data is based on CASSCF/ANO-L calculations (CAS(14,10) for C, 2tC, U, 2tU; CAS(18,13) for G, 6tG) followed by a Mulliken analysis of the difference density between ground state and excited state. The difference densities are shown in Supplementary Fig. 6.

Critical Point	Mulliken detachment	
	C	2tC
$^1n\pi^*$ min	0.51	0.68
$^1\pi\pi^*$ min	0.36	0.35
S_0/S_1 semiplanar	0.54	0.92
S_0/S_1 oop-NH_2	0.06	0.39
S_0/S_1 ethylenic	0.16	—
Critical Point	Mulliken detachment	
	U	2tU
$^1n\pi^*$ min	0.62	0.81
$^1\pi\pi^*$ min	0.09	0.63
S_0/S_1 oop-Y (Y=O/S)	0.03	0.75
S_0/S_1 ethylenic	0.75	—
Critical Point	Mulliken detachment	
	G	6tG
$^1n\pi^*$ min	0.68	0.77
$^1\pi\pi^*$ min	0.36	0.35
S_0/S_1 oop-Y (Y=O/S)	0.07	0.64
S_0/S_1 oop-NH_2	0.03	0.20

Supplementary Table 6: Levels of theory used in the calculations. In column "State-Averaging", "3S or 3T" means two separate calculations, averaging over 3 singlet states in the first one, and averaging over 3 triplet states in the second one, as applicable. "4S+2T" means simultaneously averaging over 4 singlets and 2 triplets. "various" refers to the minimum number of roots required for each individual calculation (SS-CASSCF for S_0 minimum, SA2 for S_1 minimum, SA3 for S_2 minimum, etc.). "MRCIS" is shorthand for MRCIS(6,5)/cc-pVDZ, orbitals based on SA(4S+2T)-CASSCF(10,8)/cc-pVDZ.

Step	Method	Basis Set	State-Averaging	Program	Used In
		— Tautomers —			
Opt/Freq	RI-MP2	cc-pVTZ	—	Orca	Supp Tab 7
Energies	RI-CCSD(T)	cc-pVTZ	—	Orca	Supp Tab 7
		— Excited-state optimizations —			
S_0 minimum	RI-MP2	cc-pVQZ	—	Orca	Supp Tab 1, Supp Fig 2
Minima	CASSCF(14,10)	ANO-S	various^c	Molcas	Supp Tab 2, Fig 3
CoIn, MECP	CASSCF(14,10)	6-31G*	various^c	Molpro	MEPs
MEPs	CASSCF(14,10)	ANO-S	various^c	Molcas	Fig 4
Min, CoIn, MECP (reopt.)	MS-CASPT2(14,10)	ANO-L	3S or 3T	Molcas	Supp Tab 3, Fig 4
Min, CoIn, MECP	MRCIS	cc-pVDZ	4S+2T	Columbus	Supp Tabs 1, 3<br>Supp Figs 2, 5
		— Excited-state energies —			
Ground state spectrum	MS-CASPT2(14,10)	ANO-RCC-VQZP	9S or 8T	Molcas	Supp Tab 1, Supp Fig 2
	MRCIS	cc-pVDZ	4S+2T	Columbus	Supp Tab 1, Supp Fig 2
Transient spectra	MS-CASPT2(14,10)	ANO-L	15S or 15T	Molcas	Supp Tab 2, Fig 3
Min, CoIn, MECP	MS-CASPT2(14,10)	ANO-L	3S or 3T	Molcas	Supp Tab 3, Fig 4
	MRCIS	cc-pVDZ	4S+2T	Columbus	Supp Tab 3, Supp Fig 5
		— Dynamics —			
Sharc simulations	MRCIS	cc-pVDZ	4S+2T	Columbus	Supp Tab 4, Fig 5
		— Difference Densities —			
C, 2tC, U, 2tU	CASSCF(14,10)	ANO-L	4S	Molcas	Tab 1, Supp Fig 6, Supp Tab 5
G, 6tG	CASSCF(18,13)	ANO-L	4S	Molcas	Tab 1, Supp Fig 6, Supp Tab 5

Supplementary Table 7: Energies, populations and dipole moments of 2-thiocytosine tautomers. E_{SP} is the RI-CCSD(T) single point energy, ZPE is the RI-MP2 zero-point energy, E_{rel} is the energy relative to the most stable tautomer, and μ is the permanent dipole moment (at CCSD(T) level). The temperature assumed for the calculation of the populations is 298 K. The geometries and ZPE were computed at the RI-MP2/cc-pVTZ [11–13] level of theory and the single-point energies at the RI-CCSD(T)/cc-pVTZ level of theory (in vacuum and in water COSMO [14]). These calculations were performed with the Orca 3.0 package. [15]

Tautomer	E_{SP} (E_h)	ZPE (E_h)	E_{total} (E_h)	E_{rel} (eV)	E_{rel} (kcal/mol)	Population	μ (Debye)
			— Gas Phase —				
TC1	−717.970916	0.097153	−717.873764	0.222	5.12	0%	7.24
TC2a	−717.975696	0.093771	−717.881926	0.000	0.00	57%	3.59
TC2b	−717.975446	0.093792	−717.881654	0.007	0.17	43%	4.29
TC3	−717.959488	0.096858	−717.862630	0.525	12.10	0%	8.29
TC4	−717.970505	0.097514	−717.872991	0.243	5.60	0%	7.93
			— COSMO (water) —				
TC1	−718.003190	0.097295	−717.905894	0.000	0.00	100%	
TC2a	−717.991223	0.093254	−717.897970	0.216	4.97	0%	
TC2b	−717.991339	0.093288	−717.898051	0.213	4.92	0%	
TC3	−717.994842	0.097709	−717.897132	0.238	5.49	0%	
TC4	−717.992620	0.097743	−717.894877	0.300	6.91	0%	

Supplementary Note 1: Simulated Ground State Spectrum

A ground-state absorption spectrum of 2-thiocytosine was simulated as a sum of Gaussians centered at the vertical excitation energies obtained from MS-CASPT2 (Supplementary Fig. 2a). Notable contributions to the spectrum come from the S_2, S_4, S_6 and S_8, which are of $\pi_S\pi^*$ or $\pi\pi^*$ character and show significant oscillator strengths (see Supplementary Table 1). Supplementary Fig. 2b shows the simulated absorption spectrum calculated with MRCIS with 4 excited states based on 4000 geometries from a Wigner distribution. Also shown is the energy range for selecting initial conditions for the dynamics simulations (grey box), in accord with the experimental conditions.

Similar to the MS-CASPT2 spectrum, the MRCIS spectrum reveals S_2 and S_4 as the main contributing states. However, compared to the experimental and the MS-CASPT2 spectrum, the MRCIS spectrum shows a very broad and red-shifted S_2 absorption band (between 290 and 400 nm) and an S_4 band with the correct width but shifted to too high energies (242 nm instead of 268 nm). The main reason for these deviations is the less complete description of electronic correlation in the MRCIS calculations. In particular, due to the small reference space employed, our MRCIS setup does not describe well higher excited states; however, the lowest excited states, which are the relevant states for the dynamics, are reasonably well described. Indeed, the focus of this work is the excited-state dynamics after UV-A/UV-B excitation leading to the population of S_2, and hence the inaccurate description of the S_4 state is not relevant.

Supplementary Note 2: Critical Points

Supplementary Table 3 collects energies of the relevant critical points of the excited-state potential energy surfaces (PESs), including minima, conical intersections (CoIns), and singlet-triplet minimum-energy crossing points (MECPs), on the MS-CASPT2 and MRCIS levels of theory for comparison.

Compared to the MS-CASPT2 results, the optimization of the critical points at the MRCIS level of theory, which was employed in the dynamics simulations, yields a slightly different picture (see energies in Supplementary Table 3 and Supplementary Fig. 5). Energy deviations (relative to MS-CASPT2) for all singlet and triplet minima and critical points are between 0.1 and 0.2 eV, with the notable exception of the semiplanar S_1/S_0 CoIns. At the MRCIS level of theory, the relaxation mechanism after excitation to the S_2 in the Franck-Condon region begins with a decay of the system to the ${}^1\pi_S\pi^*$ (S_2) minimum at 3.01 eV. The ${}^1n_S\pi^*/{}^1\pi_S\pi^*$ is located at 3.14 eV, resulting in a 0.13 eV barrier for $S_2 \rightarrow S_1$ interconversion. From this CoIn, the ${}^1n_S\pi^*$ (S_1) minimum can be reached at 2.76 eV. Ground state relaxation is predicted to be possible from three different CoIns at energies of 3.47, 3.63 and 3.68 eV—giving a minimum barrier of 0.61 eV. ISC to the triplet manifold is predicted to occur around the ${}^1n_S\pi^*/{}^3\pi_S^*\pi^*$ MECP at 2.83 eV (0.07 eV above the ${}^1n_S\pi^*$ minimum). In agreement with the MS-CASPT2 calculations, the lowest-energy triplet minimum is the ${}^3\pi_S\pi^*$ minimum at the MRCIS level of theory.

Supplementary Note 3: Geometry Data of Critical Points

In the following we present all geometries used in Fig. 4 in the main manuscript (optimized on CASSCF level of theory, see Supplementary Table 6).

```
13
  S0 Minimum
N +3.533273 +3.551459 -2.072505
C +2.225133 +3.342086 -2.349205
H +1.577278 +3.162683 -1.504718
C +1.785903 +3.363210 -3.635321
H +0.750385 +3.193983 -3.880958
C +2.791320 +3.602581 -4.634191
N +2.456170 +3.567833 -5.947167
H +3.159487 +3.850901 -6.607831
H +1.494976 +3.571747 -6.232838
N +4.066211 +3.840554 -4.352504
C +4.488756 +3.862179 -3.063904
H +3.875844 +3.563335 -1.123794
S +6.034968 +4.230744 -2.568172

13
  S1(npi*) Minimum
N +3.528057 +3.697829 -2.054831
C +2.196922 +3.315392 -2.299455
H +1.579122 +3.083823 -1.452035
C +1.806226 +3.277415 -3.646091
H +0.785756 +3.023873 -3.893672
C +2.727192 +3.550403 -4.637680
N +2.458095 +3.503051 -6.006251
H +3.165042 +3.983426 -6.543240
H +1.528608 +3.797604 -6.263812
N +4.096845 +3.730629 -4.351257
C +4.415333 +3.810726 -3.097042
H +3.869092 +3.862412 -1.122510
S +6.083415 +4.066719 -2.605234

13
  S2(pipi*) Minimum
N +3.545661 +3.624027 -2.040796
C +2.186990 +3.331484 -2.301288
H +1.542338 +3.188780 -1.454753
C +1.805209 +3.320047 -3.647600
H +0.776111 +3.108382 -3.899321
C +2.737465 +3.560019 -4.636299
N +2.491270 +3.460534 -6.007481
H +3.140834 +4.014694 -6.546747
H +1.535957 +3.646007 -6.273482
N +4.091109 +3.811275 -4.336188
C +4.405504 +3.830655 -3.082142
H +3.897533 +3.641886 -1.099058
S +6.083721 +4.165488 -2.647956

13
  T1(pipi*) Minimum
N +3.547144 +3.591135 -2.053086
C +2.190412 +3.326781 -2.289430
H +1.538543 +3.243318 -1.440960
C +1.803981 +3.326878 -3.652582
H +0.774560 +3.121134 -3.908331
C +2.733596 +3.558448 -4.634393
N +2.495569 +3.461710 -6.000707
H +3.144599 +4.011208 -6.543796
H +1.538191 +3.600324 -6.283933
N +4.079314 +3.857921 -4.347002
C +4.415085 +3.848811 -3.070547
H +3.923881 +3.578774 -1.119899
S +6.054829 +4.176861 -2.628445

13
  T2(npi*) Minimum
N +3.527597 +3.701936 -2.027528
C +2.184601 +3.331819 -2.303473
H +1.534364 +3.194741 -1.459630
C +1.807602 +3.302594 -3.640920
H +0.785091 +3.059691 -3.892163
C +2.737971 +3.559862 -4.634362
N +2.488223 +3.464090 -6.007278
H +3.163667 +3.987790 -6.544958
H +1.545028 +3.703170 -6.274463
N +4.086754 +3.802122 -4.324747
C +4.396447 +3.841736 -3.076309
H +3.894033 +3.599901 -1.096336
S +6.088325 +4.153847 -2.690938

13
  S1(npi*)/S2(pipi*) CoIn
N +3.554550 +3.587656 -2.043624
C +2.181685 +3.352189 -2.301423
H +1.551585 +3.155167 -1.455323
C +1.797964 +3.332628 -3.644484
H +0.768185 +3.124767 -3.895296
C +2.730709 +3.565480 -4.634402
N +2.493352 +3.456971 -6.005081
H +3.153259 +3.997050 -6.546143
H +1.541715 +3.639682 -6.285031
N +4.081422 +3.832175 -4.332255
C +4.403131 +3.833628 -3.081617
H +3.898869 +3.662744 -1.101840
S +6.083283 +4.163160 -2.646588

13
  T1(pipi*)/T2(npi*) CoIn
N +3.496169 +3.826014 -2.002564
C +2.186199 +3.349094 -2.309479
H +1.546639 +3.142567 -1.470199
C +1.813739 +3.280680 -3.629251
H +0.809761 +2.970649 -3.879937
C +2.744255 +3.578883 -4.633941
N +2.472050 +3.479884 -6.004753
H +3.187714 +3.933430 -6.554461
H +1.557341 +3.820500 -6.262949
N +4.078375 +3.794387 -4.306702
C +4.381749 +3.864002 -3.064704
H +3.870034 +3.582258 -1.097851
S +6.095679 +4.139930 -2.756318

13
  S0/S1 CoIn semiplanar 1
N +2.208365 +1.725585 -0.300734
C +0.770699 +1.746609 -0.474810
H +0.254836 +2.385111 +0.231193
C +0.386316 +1.628132 -1.907828
H -0.504885 +1.077980 -2.177521
C +1.261090 +2.006697 -2.839050
N +1.221545 +1.750683 -4.218041
H +1.569134 +2.541240 -4.743141
H +0.288539 +1.517125 -4.527934
N +2.548328 +2.599096 -2.486630
C +2.917521 +2.228115 -1.338050
H +2.654871 +1.010030 +0.261169
S +4.659193 +2.497906 -0.864962
```

```
13
  S0/S1 CoIn semiplanar 2
N +3.585134 +3.605924 -2.022385
C +2.148488 +3.844093 -2.252387
H +1.544468 +3.452936 -1.444258
C +1.780437 +3.483307 -3.646507
H +0.832136 +3.014056 -3.872836
C +2.686796 +3.701750 -4.595048
N +2.647551 +3.315373 -5.952665
H +2.980242 +4.071035 -6.537134
H +1.705823 +3.076496 -6.233382
N +4.013499 +4.250300 -4.270572
C +4.352740 +3.939213 -3.097357
H +3.885462 +2.802690 -1.480998
S +6.076896 +4.146136 -2.567622
```

```
13
  S0/S1 CoIn oopNH2
N +0.412354 +0.440880 +0.587459
C -0.942412 +0.633675 +0.331796
H -1.612367 -0.160366 +0.624070
C -1.307102 +1.774150 -0.300763
H -2.338744 +1.951150 -0.560618
C -0.230357 +2.783534 -0.452072
N +0.097795 +3.475579 +0.756015
H +0.774770 +4.206144 +0.574459
H -0.720482 +3.881423 +1.191328
N +0.989358 +2.132817 -0.945525
C +1.300890 +1.103870 -0.238017
H +0.742112 -0.374375 +1.075210
S +2.877305 +0.370250 -0.406251
```

```
13
  S1(npi*)/T1(pipi*) MECP
N +3.559357 +3.554490 -2.010884
C +2.180270 +3.368597 -2.293023
H +1.513235 +3.307688 -1.449960
C +1.801921 +3.308297 -3.625083
H +0.764921 +3.117828 -3.865450
C +2.746057 +3.496371 -4.644489
N +2.429575 +3.440996 -5.998719
H +3.221022 +3.633465 -6.591386
H +1.617484 +3.965916 -6.288919
N +4.065748 +3.861080 -4.304187
C +4.382685 +3.873001 -3.063252
H +3.931874 +3.301071 -1.112601
S +6.025554 +4.474502 -2.725643
```

```
13
  S2(pipi*)/T2(npi*) MECP
N +3.518515 +3.802662 -2.031694
C +2.192109 +3.419928 -2.317829
H +1.538498 +3.289444 -1.476177
C +1.810954 +3.332857 -3.622232
H +0.781463 +3.101772 -3.854368
C +2.753560 +3.535515 -4.638383
N +2.470264 +3.436243 -6.006704
H +3.172229 +3.895899 -6.569023
H +1.539019 +3.729092 -6.267422
N +4.100240 +3.725484 -4.328361
C +4.411492 +3.829360 -3.094600
H +3.884782 +3.645719 -1.106043
S +6.066580 +4.158554 -2.646198
```

Supplementary References

[1] Barbatti *et al.* The on-the-fly surface-hopping program system Newton-X: Application to ab initio simulation of the nonadiabatic photodynamics of benchmark systems. *J. Photochem. Photobiol. A* **190**, 228–240 (2007).

[2] Podolyan, Y., Gorb, L., Blue, A. & Leszczynski, J. A theoretical investigation of tautomeric equilibria and proton transfer in isolated and hydrated thiocytosine. *J. Mol. Struc. - THEOCHEM* **549**, 101–109 (2001).

[3] Civcir, P. U. AM1 and PM3 studies of some thio analogues of pyrimidine bases in the gas and aqueous phases. *J. Phys. Org. Chem.* **14**, 171–179 (2001).

[4] Contreras, J., Alderete, J. B. & Gnecco, J. A. Semiempirical molecular orbital calculations on the prototropic tautomerism of 2-thiocytosine. *J. Mol. Struc. - THEOCHEM* **251**, 195–204 (1991).

[5] Leś, A. & Ortega-Blake, I. Tautomerism of uracil, cytosine, isocytosine, and some of their thio-derivatives. *Int. J. Quant. Chem.* **30**, 225–237 (1986).

[6] Barysz, M., Klobukowski, M. & Leszczyński, J. Relativistic study of tautomerism and core electron binding energies of thio- and selenocytosine. *Struct. Chem.* **23**, 1293–1299 (2012).

[7] Kwiatkowski, J. S. & Leszczyński, J. Molecular structure and vibrational IR spectra of cytosine and its thio and seleno analogues by density functional theory and conventional ab initio calculations. *J. Phys. Chem.* **100**, 941–953 (1996).

[8] Latosińska, J. N., Seliger, J., Žagar, V. & Burchardt, D. V. A comparative study of the hydrogen-bonding patterns and prototropism in solid 2-thiocytosine (potential antileukemic agent) and cytosine, as studied by 1H-14N NQDR and QTAIM/ DFT. *J. Mol. Model.* **18**, 11–26 (2012).

[9] Rostkowska, H. *et al.* Infrared spectra of 2-thiocytosine and 5-fluoro-2-thiocytosine; experimental and ab initio studies. *Spectrochim. Acta Mol. Spectros.* **49**, 551–565 (1993).

[10] Rostkowska, H. *et al.* Theoretical and matrix-isolation experimental studies on 2-thiocytosine and 5-fluoro-2-thiocytosine. *Biochim. Biophys. Acta – Gene Struct. Expr.* **1172**, 239–246 (1993).

[11] Kossmann, S. & Neese, F. Efficient structure optimization with second-order many-body perturbation theory: The RIJCOSX-MP2 method. *J. Chem. Theory Comput.* **6**, 2325–2338 (2010).

[12] Dunning, T. H. Gaussian basis sets for use in correlated molecular calculations. I. The atoms boron through neon and hydrogen. *J. Chem. Phys.* **90**, 1007–1023 (1989).

[13] Woon, D. E. & Dunning, T. H. Gaussian basis sets for use in correlated molecular calculations. III. The atoms aluminum through argon. *J. Chem. Phys.* **98**, 1358–1371 (1993).

[14] Sinnecker, S., Rajendran, A., Klamt, A., Diedenhofen, M. & Neese, F. Calculation of solvent shifts on electronic g-tensors with the conductor-like screening model (COSMO) and its self-consistent generalization to real solvents (direct COSMO-RS). *J. Phys. Chem. A* **110**, 2235–2245 (2006).

[15] Neese, F. The ORCA program system. *WIREs Comput. Mol. Sci.* **2**, 73–78 (2012).
